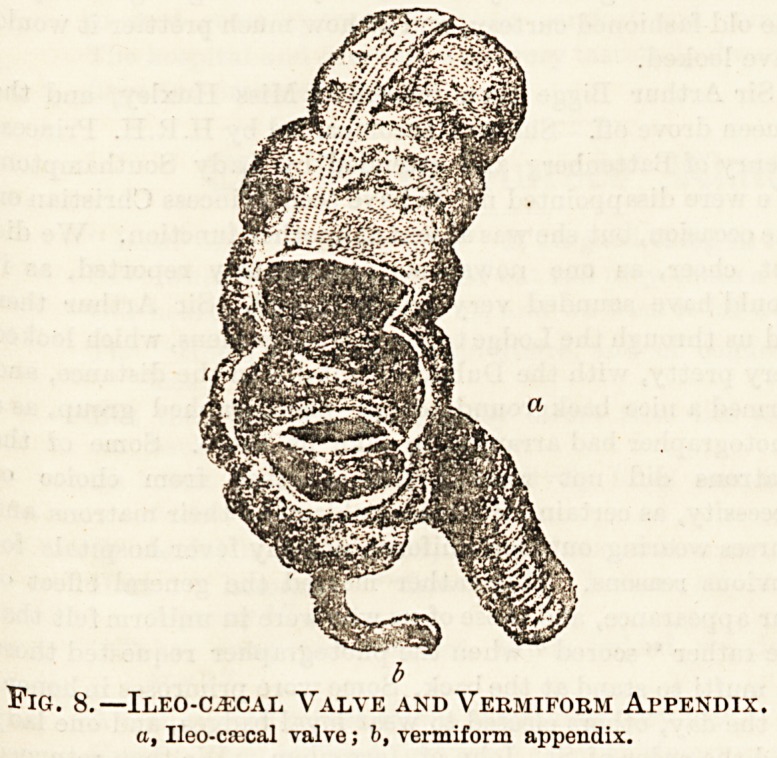# "The Hospital" Nursing Mirror

**Published:** 1900-04-28

**Authors:** 


### "The Hospital" Nursing Mirror.

Being the Nursing Section of "The Hospital."

[Contributions for this Section of "The Hospital" should be addressed to the Editor, The Hospital, 28 & 29, Southampton Street, Strand, London, W.C., and should have the word "Nursing" plainly written in left-hand top corner of the envelope.]

### Notes on News from the Nursing World.

### THE QUEEN'S VISIT TO THE MATER MISERICORDIÆ HOSPITAL.

Not only the Princess Christian, but the Queen herself has paid a visit to the Mater Misericordiæ Hospital at Dublin. The proceedings on Tuesday were of a very enthusiastic character. A large crowd assembled in the neighbourhood of the hospital, and the building was gaily decorated with trophies and flags, the Irish national flag streaming from the top. The group on the steps included the sisters of mercy and the nurses in grey uniforms. Sir Christopher Nixon and the medical staff, with the lady superintendent of the nursing staff, were in attendance. At the termination of her visit, the Queen, addressing Sir Christopher Nixon, said: "I am very greatly pleased with my visit. This is a very large hospital. How many beds have you in occupation?" Sir Christopher Nixon replied: "There are, your Majesty, 340; but quite 500 souls sleep under the roof every night; and the hospital is managed exclusively by sisters of mercy, and supported by voluntary contributions."

### NIGHTS AT MARITZBURG.

An Army Reserve Sister writes to us from Maritzburg: "I sleep in a tent. Certainly the arrangement has its disadvantages. Little beasties crawl over everything. Small animals seem to have such a power over you; in fact, they are absolute lords of creation. A flea can disarrange all one's plans and avoid execution by his wily jumps; and the plague of lice must have been the worst of all for the Egyptians. One gets very little air in the tent, and that at the expense of having one's bare feet seen from underneath the canvas. Our sheets get very dirty from the earthen floor, and the blanket is like a black horse-rug. The last few nights we have had a beautiful full moon. This morning, while everything was bathed in the moonlight, right away in the east the sun was rising, and the effect was lovely. The town lies just below us, and behind are the hills. In the afternoon, if it is fine, the heat is intense, and it is quite impossible to sleep; but I enjoy the mornings."

### THE RETURN OF THE "MAINE."

The "Maine" arrived unexpectedly at Southampton on Sunday afternoon. Lady Randolph Churchill states that everything went off splendidly, and that there was not a single hitch nor disagreement with anyone. Being asked why the vessel had come home, Lady Randolph replied that since the relief of Ladysmith the hospitals had become terribly congested, and all the hospital ships were full. The progress of the patients on board has been most satisfactory, and Lady Randolph says that the best feeling prevailed among all on board. Three deaths occurred during the voyage, but the cases were from the first regarded as desperate.

### NURSING THE SICK SOLDIER IN INDIA.

The strictures passed on military nursing by Dr. Groves in his report on the subject of Nursing in Military Hospitals to the British Medical Association have excited much interest in the Indian press, and redirected public attention to some articles on the "Sick Soldier in India," which were published in a Punjab newspaper, and referred to in our columns a short time ago. Whatever Surgeon-General Hamilton may have to say in favour of the R.A.M.C. orderlies, who are at least well-trained male nurses, he could have little to urge on behalf of the regimental nursing orderlies and native attendants of the Indian Army Hospital Corps who take their place in India. The regimental orderlies are well-meaning men, but they are soldiers very imperfectly trained in nursing duties, and are liable to recall to the barrack square, riding school, or gun park at the whim of their regimental commanding officer. The native attendants are camp followers of the lowest order, and in Northern India rarely even understand English. The nurses of the Indian Army Nursing Service are unfortunately numerically very few, and are only employed in the largest stations. In short, the nursing of the sick soldier in India imperatively claims attention.

### NURSES AND THE WELSH LANGUAGE.

It has been decided by the Guardians of the Bangor and Beaumaris Union to abandon the policy of making a knowledge of Welsh essential to candidates for the post of head nurse in the workhouse hospital. This course has not been taken without good reason; for, although the appointment has been advertised for three months, no suitable candidate has yet presented herself. There is a good deal of force in the contention that temperament is more important than language, and it appears that, though the former head nurse could not speak Welsh, she discharged her duties in a most satisfactory and successful manner. An attempt was made to secure the post for the assistant nurse, but it was pointed out that the Local Government Board would not be likely to approve the selection of an untrained person. Moreover, now that proficiency in Welsh is no longer indispensable, the Guardians will probably have little difficulty in obtaining the services of a fully qualified head nurse.

### THE ALTERATIONS AT ST. GEORGE'S HOSPITAL.

Most of the nurses' bedrooms at St. George's Hospital are finished and in occupation, but the remainder of their quarters and the new kitchen are still in the possession of the builders and engineers. The time and strength of the matron have been severely taxed in order to keep the nursing organisation working smoothly in face of so many difficulties; but her health has greatly benefited by a rest of some weeks, and she is now at her post again.

### THE BIRMINGHAM DISTRICT NURSING SOCIETY.

In addition to the annual report of the Birmingham District Nursing Society, the House Committee have published their first annual report of the new nursing home. It is stated that the advantages of the home have shown themselves continually during the year, both in the greater proximity of the districts, which has enabled the nurses to get through considerably more work, and in the personal comfort afforded to the nurses by the healthy situation of the house and the cosy and suitable manner in which it has been furnished. Seven districts are being worked from this home, which is capable of accommodating nine nurses, and it appears that only lack of funds prevents the committee from filling it. The number of nurses on the staff of the society is sixteen, and here again want of adequate income hinders the committee from making a very necessary increase. The Birmingham nurses are doing, as the report shows, such excellent work that the appeal for sufficient support to fully utilise the two homes can scarcely fail to be successful. Accompanying the report are two admirable pictures of the superintendent and staff of nurses at each home, which will doubtless have the effect of increasing the interest in their operations.

### IRISH GUARDIANS AND THE "NURSING INCUBUS."

The Guardians of the Nenagh Union are very angry at the Irish Local Government Board for insisting upon the maintenance of an adequate nursing staff, which they call a "nursing incubus." To quote their own words, "The poor ratepayers are being beggared, and if something is not speedily done to prevent this ruinous extravagance many of them will be seeking admission to the workhouse." The last grievance is that the Guardians have been called upon to provide a nurses' home, which they evidently do not consider necessary. They have a perfect right to give expression to their views on the question of economy, but we hope that they are not trying to visit the supposed transgressions of the Local Government Board on the heads of the nurses. They have not only flatly refused some of the latter leave of absence, but they have peremptorily declined to make allowances in lieu of rations to others who complained that the workhouse baked bread was not good enough.

### DEARTH OF MENTAL NURSES IN DUNDEE.

Even at such an admirably managed institution as the Dundee Royal Asylum a difficulty is experienced in securing suitable persons for training as nurses. At the quarterly court of directors Mr. Cameron said he recently observed that there were 300 applicants for permission to act as probationers at the Royal Dundee Infirmary, and he seemed to think it strange that there was not the same desire to enter the service of the asylum. But the truth of the matter is that until the attendants at asylums, not only in Scotland, but also throughout the kingdom, are drawn from a higher social plane than they have been hitherto the difficulty will remain. No doubt the inducements which the managers of the Dundee Asylum have decided to offer by increasing the scale of remuneration and improving the dietary, may tend in that particular case to effect the result desired, but the question is one that requires to be dealt with generally.

### NEVER HAD A NURSE BEFORE.

It is satisfactory to learn from a speech at the annual meeting of the Carlisle District Nursing Association that the committee have been able to stretch their beneficent arms over the whole of the city, and that now there is no poor person whom a medical attendant thinks requires a nurse who cannot have the benefit of her aid. Chancellor Prescott said he felt a great deal in regard to that matter, because since he last attended this annual meeting, after a considerable number of years of life, he had fallen ill for the first time, and "he must say that his experience of the success with which a nurse can make you do what you don't want to do, and make you take what you don't want to take, and the skill with which she can take things off you and put them upon you before you know where you are, was a complete revelation." Another member of the committee said they used to think that operations could only be conducted in the infirmary, but now he was glad to say a great many were performed in the houses of the people, and there the nurses, while gaining admirable experience, rendered valuable assistance.

### MARTYRS TO DUTY.

The chapel at the Middlesex Hospital has lately been beautifully decorated after the designs of the late J. L. Pearson, R.A., the cost being defrayed by a munificent legacy from a former Broderip scholar—Mr. Kenneth Lawson, M.R.C.S. The dedication is as follows: "To the glory of God and in memory of those faithful servants of the hospital who, since its original foundation in the year of our Lord MDCCXLV., have died from sickness contracted in the discharge of their duty, and of the friends of the hospital who have conspicuously devoted their time and energies to its welfare, this chapel is erected, MDCCCLXXI." A feature of interest in the chapel is a little group of tablets recording the names of devoted nurses who have laid down their lives for their patients; while on another wall are inscriptions to the memory of officers, benefactors, and medical men who were martyrs to their profession.

### PROGRESS OF NURSING IN FLINTSHIRE.

The Flint County Council announce that, with "the view of providing systematic and skilled nursing in the county, they are prepared to offer a number of scholarships for the training of women in village nursing. The scholarships, it is understood, will each be of the value of £40 per annum, and the conditions are that the candidate is an inhabitant or native of the county, between 18 and 35 years of age, that she completes the full course of training designated by the Council, and that when trained she will act as nurse in the county for not less than three years, provided she receives a salary of not less than £50 per annum. In proof of the need of such a scheme, it is stated that at present there are only 26 nurses employed in the whole of the county, 15 of these being in the town of Rhyl; while there are at present in the county no less than 29 parishes, with a population of nearly 40,000, with no nurses at all and no hospital or other accommodation." It has also been decided to form a County Nursing Association. Dr. Eyton Lloyd has suggested that the association should have a certain number of thoroughly trained women, with a number of inferior nurses to work under their supervision. His idea is that groups of parishes could be formed, and that in each group there could be a fully qualified nurse to go round to the serious cases, while the less serious cases could be taken by the assistant nurses. Dr. Lloyd properly contends that a person, trained for six months, would be useless in a serious case of pneumonia, which of necessity is a disease that had to be treated at home.

### WEST BROMWICH DISTRICT NURSES' ASSOCIATION.

It is satisfactory to learn that district nurses are becoming increasingly popular in the Black Country. As a Jubilee gift, a pretty little home was built for three nurses at Wednesbury, from whence excellent work has been going on ever since, and now its neighbour, West Bromwich, has just held its first annual meeting, at which the report, recording 583 cases nursed and 10,232 visits paid during the nine months of its existence, was agreed to. Early last year an experienced district nurse offered her services for work in the Midlands to the Bishop of Lichfield, and he put her in communication with the Rural Dean of West Bromwich, knowing well the immense number of poor in that town. A public meeting was held, with the Bishop in the chair, and the offer was formally made to West Bromwich. It was joyfully accepted, and a provisional committee formed to raise the necessary funds to carry out the scheme. In May a second nurse was engaged, and in November a third, the work spreading rapidly as the people began to realise the blessing and comfort of skilled nursing. Money to rent and furnish a hired house for the nurses was raised by subscriptions and a series of dramatic entertainments given by a resident in the town, the hon superintendent being lent a little house adjoining. An appeal is now being made for funds to provide one or two more nurses and a house where they could live altogether. Councillor Akrill offered to build a permanent home to hold five nurses, Lord Dartmouth having previously offered a site. Messrs. R. Hudson and Co. gave £250, and Mr. Archibald Kenrick £100, towards the £1,000 that will be required for the endowment. If the other manufacturers in West Bromwich will now make up the remaining £650, this good work, so admirably commenced, will be placed upon a firm basis.

### KIMBERLEY NURSING ASSOCIATION.

There is a Kimberley not in South Africa, and we are glad to learn from the first annual report of its Nursing Association that the residents of the district appreciate the experiment which was started in February of last year. Since then, Nurse Boden has paid upwards of 1,200 visits in Kimberley and four other Nottinghamshire villages; the financial condition of the association is satisfactory, and through the liberality of the subscribers there is a balance of £62 in hand. This is after paying all expenses, including £11 for a cycle—a most necessary adjunct of an association whose representative has to cover long distances.

### TRAINING AT SANATORIA.

In view of the fact that the Exeter City Council have just decided to train their own probationers at their sanatorium, it may be well to point out that such training would not be accepted as sufficient at the principal hospitals. No doubt probationers who are trained at small sanatoria, where they may be taught either for purposes of economy or with the notion of securing a supply, may be pretty sure of retaining employment, but they have no prospect of making any advance in other branches of their profession.

### AN ENGLISH DISPENSARY IN SYRIA.

"Three days a week is our dispensary open," writes a correspondent, "and some idea of the use the natives make of it may be realised by the statement that from 20 to 50, or even 60, people come for medicine each 'pharmacy' day." Our correspondent continues: "The house used for the dispensary was originally a prison, and has numerous dark cells on one side of the large entrance-hall. We have an English qualified dispenser (a nurse), and her 'shop' is kept in first-rate order, and is quite up-to-date. The Syrian doctor sees his patients in a little consulting-room. When necessary he writes a prescription, and gives them a ticket, for which they pay the nominal sum of two metlicks (about 1¼d.) This they hand with a bottle through a little kind of office door to the dispenser, or to her assistant and interpreter. The patient then walks to another door, and waits till the "domer" (medicine) is ready. The patients have to provide their own bottles, and some of the vessels brought are very curious, many being pretty and quaint vases or decanters, far too nice for the purpose. Occasionally the bottles are very dirty, and sometimes they smell strongly. The other day one had the unmistakable odour of paraffin, and upon being questioned the woman who brought it said she had only used it for 'gas'—petroleum!"

### THEN AND NOW.

"A short time ago," says a nurse in the provinces, "I had an amusing encounter with a sister of the immortal 'Sairey Gamp.' She was seated opposite to me in a railway carriage, and feeling I was being stared at I looked up, when she at once commenced conversation. 'Humph! I suppose you call yourself a lady nurse?' Then, giving me no time to deny the impeachment, she went on with a regular tirade on the subject of modern nurses. 'I've no patience wi' you lady nusses, dressing yerselves up like widdies, and taking the bread out o' poor folks' mouths.' There was a good deal more to the same effect, making me feel rather uncomfortable, and causing some amusement to a couple of fellow-travellers. In the course of conversation I discovered that the woman whose ire my costume had aroused had been a nurse in my own hospital thirty years ago, and greatly resented the modern innovations."

### SHORT ITEMS.

Nursing Sister E. A. Chapple has been permitted by the Secretary of State for India to retire from the Indian Army Nursing Service.—The annual meeting of the Clapham Maternity Hospital will be held at the hospital, Jeffrey's Road, on Wednesday, May 2nd. Dr. Helen Webb will take the chair at half-past three p.m.—Some very stirring lines, addressed "to the Nurses in South Africa," have been written by Mr. Albert Mills, a bed-ridden patient, who is nursed by the nurses of the Birmingham District Nursing Society.

### Lectures on Nursing for Probationers.

By E. MACDOWEL COSGRAVE, M.D., &c., Lecturer to the Dublin Metropolitan Technical School for Nurses.

### IV.—THE ABDOMEN.

THE abdomen is the largest cavity of the body; it separates the chest from the pelvis, and contains the greater part of the digestive canal, the liver, pancreas, spleen, kidneys, and other structures. It is bounded above by the diaphragm, which arches up behind the lower ribs; behind by the five lumbar vertebræ and the muscles of the back; at the sides by the lower ribs, and lower down by three layers of muscle; in front by the right and left rectus muscles, which run from the sternum to the pubes, and are joined to the lateral muscles, and to one another, by strong layers of fibrous

FIG. 6.—TRANSVERSE SECTION OF ABDOMEN.

tendons; below by the brim of the pelvis, across which there is no partition, the pelvic and abdominal organs being in contact. The three muscles at the side are the external oblique, whose fibres run downwards and forwards; the internal oblique, running downwards and backwards; and the transversalis, running across.

For convenience of description the abdomen is divided into nine regions. To mark out these regions four lines must be drawn—two down and two across. One line is drawn at each side from the cartilage of the eighth rib to the middle of Poupart's ligament (which runs across the top of the thigh from the pubes to the ilium), and two are drawn transversely, one running round the body at the level of the cartilage of the ninth rib, and one lower down at the level of the crest of the ilium. The regions are named :—

| | | |
|---|---|---|
| Right Hypochondriac Region | Epigastric Region | Left Hypochondriac Region |
| Right Lumbar Region | Umbilical Region | Left Lumbar Region |
| Right Iliac Region | Hypogastric Region | Left Iliac Region |

When food is taken into the mouth it is masticated, it is then swallowed, passing into the pharynx and down the œsophagus through an opening in the diaphragm into the stomach. The stomach is a bag-like dilatation. It is situated in the upper part of the abdomen in the left hypochondriac and epigastric regions. The food enters by the cardiac opening, so called because it is situated just behind the apex of the heart; the other opening, through which food passes out, is called the pyloric opening, because it is cl sed by a ring of muscular fibres called the pylorus, or "gate-keeper"; it is situated to the right of the epigastric region.

The walls of the stomach and digestive canal are largely composed of involuntary muscular fibres, and by the contraction of these muscles the food is kept moving about, mixing with the gastric juice and becoming digested. When the contents of the stomach become fluid the pylorus relaxes and allows them to pass out into the small intestine, which to the length of some 20 feet is coiled up in the centre of the abdomen, being suspended to the front of the spine by the frill-like mesentery which conducts the blood-vessels, &c., to the intestine; by the contraction of the muscular walls the contents are gradually passed on through the tube. The small intestine is divided into three parts, the duodenum, jejunum, and ilium; into the first the bile from the liver and the pancreatic juice flow by a common duct, mixing with the food and helping to digest it. The small intestine ends in the right iliac region; it opens into the side of the cæcum, which is prolonged downwards into a small process, the vermiform appendix, passing upwards into the colon or large intestine, which passes upwards, then across, and then down to the left iliac region, where it passes through the sigmoid flexure into the rectum, which is situated in the pelvis. These three parts are called the ascending, transverse, and descending colon.

The liver is a large organ weighing some three or four pounds; it is situated in the right hypochondriac and epigastric regions. It forms bile which is stored in the gall-bladder, which holds about an ounce. The liver also acts as a temporary storehouse for food.

The spleen is deep down in the left hypochondriac region, and is soft, so cannot be felt unless when hardened or enlarged. From the spleen across to the duodenum runs the pancreas or sweet-bread; it forms the pancreatic juice.

There are two kidneys situated at the back of the abdomen in the lumbar regions; they are outside the peritoneum, and reach from the eleventh rib nearly to the crest of the ilium. They are important organs of excretion drawing off waste material from the blood. From each a ureter runs to the bladder, which is in the pelvis, rising, when distended, into the abdomen.

The aorta enters the abdomen through an opening in the diaphragm, and runs

FIG. 7.—THE ALIMENTARY TRACT.

*cs*, Fundus of stomach; *s*, greater curvature of stomach; *œ*, œsophagus; *sp*, spleen; *py*, *p*, pyloric end and pylorus of stomach; *d*, duodenum; *i*, small intestine; *l*, liver; *gb*, gall bladder; *va*, vermiform appendix; *c*, cæcum; *ac*, ascending colon; *tc*, transverse colon; *dc*, descending colon; *ic*, sigmoid flexure; *r*, rectum; *a*, anus.

on the bodies of the vertebræ—a little to the left—until it reaches the middle of the umbilical region, where it divides into two branches, which supply the lower limbs with blood.

The abdomen should be resonant when percussed. If any part of the digestive canal is distended with flatulence it may be too resonant (tympanitic). Dulness of part of the abdomen may arise from many causes, such as lodgment of fæces in the descending colon, enlargement of the liver or spleen,

FIG. 8.—ILEO-CÆCAL VALVE AND VERMIFORM APPENDIX.

*a*, Ileo-cæcal valve; *b*, vermiform appendix.

dropsy into the peritoneum (ascites), new growths—for example, cancer; enlargement of some pelvic organ—for example, pregnancy, ovarian tumour, distended bladder.

Hernia is a tumour formed by some part of the contents of the abdomen—generally part of the intestine—pushing out of the abdomen. It generally gets through the opening through which blood-vessels or other structures pass out.

### East London Nurses at the Mansion House.

BY A SPECTATOR.

THE "Saloon," as the room containing the beautiful picture of Lady Jane Grey is called, was very fairly filled on Tuesday afternoon, when the Lord Mayor, preceded by a magnificently arrayed personage in gold lace and cocked hat, was announced. He was warmly received, and his benevolent face and manner gave a homely character to the proceedings, which would otherwise have been almost too civic. Several speakers, notably the chairman of the East London Nursing Society, himself an old volunteer, alluded to the part recently taken by Sir Alfred Newton in the raising of the City Corps, and a cutting from the *Daily Graphic*, framed and hung in the room, reminded one still further of wars and rumours of wars.

But the subjects of interest on this occasion were not army nurses, but the thirty-one East London nurses who, without glory and without excitement, plod on year after year, "putting people straight," and teaching their patients the principles of cleanliness and good nursing. As one of the speakers suggested, it is an excellent thing to teach children in the schools the laws of health, and when this instruction is followed up by the district nurse in their own homes, it becomes invaluable.

### "A BATH WHETHER HE WANTED IT OR NOT."

The Rev. W. H. Davies, rector of Spitalfields, told a suggestive story of a friend of his, who was asked the other day by a working man, in great surprise, "Does your baby have a bath every day?" "Yes; I'm not sure that he doesn't have two." "But whether he wants it or not?" "Certainly." "Why, of course," the labouring man's wife struck in, "don't you know his mother's a lidy?" But why should cleanliness be confined to "lidies" and gentlemen! Why not every baby have his bath whether he wants it or not? And this is a most important part of the work of a district nurse—to teach people the need of soap and water. But how pale and tired some of the thirty-one nurses looked! One would have liked to pack them off to the seaside before letting them go back to Whitechapel, or Bethnal Green, or Shadwell.

### MORE NURSES WANTED.

Thirty-one nurses to a population of 337,800 is a pitiably inadequate number, is it not? No wonder they are often overworked, especially when, as sometimes happens, a nurse is "lent" at the urgent request of the doctor, to attend a patient in a district which cannot afford a nurse of its own, and this in addition to her already heavy day's work. Mr. Davies, speaking evidently from intimate knowledge of the people among whom he works, drew a simple but pathetic picture of the mother of a family, ill in bed, but not ill enough for the hospital, the daughter obliged to leave early to go to work, and the neighbour, very willing, but quite unable to find time to go in and brush the patient's hair. A little touch of nature that more than many sensational stories could have done made one realise what it must be not to have the common comforts of illness.

### AN ANGEL UNAWARES.

Not the nurse, be it understood! She is always amused to hear herself so romantically described, and pictured smoothing pillows and wiping brows with eau-de-cologne. No, the angel unawares, who was perhaps, as Prebendary Jones, the society's chairman, hoped, in the room, is a person with a large balance at the bank, and an overwhelming desire to draw it in favour of the East London Nursing Society. Indeed, want of funds was the burden of all the speeches on Tuesday afternoon, and it was a pity that there were not more outsiders among the audience, for the burden of support must fall pretty much upon the same shoulders year by year.

A proposal made by the Lord Mayor as to approaching those of the livery companies whose names do not appear in the subscription list will, probably be acted upon; and it was also suggested that the Jewish Board of Guardians in the Spitalfields district might be glad to help, especially as quite as many Jews have been nursed during the year in that district as Gentiles. If the poor parishes themselves could only raise a small fixed sum, it was thought that such societies as the Bishop of London's Fund and the Church Pastoral Aid might meet them half-way; but the clergy are for the most part poor, and they are afraid to risk an additional burden. It is touching to think of the £20 subscribed by the poor East-enders themselves out of gratitude for what the nurses have done for them—"I was sick, and ye visited me."

### "AN EDUCATIONAL FORCE."

It must have been very encouraging to the nurses to listen to the thoughtful remarks of the Rev. H. V. Le Bas, who moved the adoption of the report and balance-sheet. He described the East London Nursing Society as a truly educational force. It was doing a great deal of work with comparatively small funds. It was a society which did not pauperise. The nurse did not go to people with shillings and sixpences, not knowing whether they needed them or not; it was not a society which was imposed upon, for people did not feign illness in order to have the pleasure of being nursed. It was a work which gave a higher tone to the lives of those among whom the nurses worked, and brought sweetness and light into their homes.

### The Queen and the Dublin Nurses.

On Thursday last week, by command of the Queen, the matrons of the Dublin hospitals assembled at the Viceregal Lodge, and were received by Colonel Carrington, and subsequently inspected by Her Majesty. The matrons formed a line in front of the Lodge, and were headed by three senior matrons, Miss Huxley, Mrs. Kildare-Treacy, and Miss MacDonnell. Miss Huxley was presented to Her Majesty. An illuminated address from the matrons and nurses will be sent to Her Majesty in a few days through Miss Huxley and Mrs. Kildare-Treacy.

The following are the number of nurses under the supervision of the matrons who were present: Mrs. Kildare-Treacy, 150 nurses, City of Dublin Nursing Institution; Miss Huxley, 30 nurses, Sir Patrick Dun's Hospital; Miss MacDonnell, 40 nurses, Richmond Hospital; Miss Lyons, 24 nurses, Meath Hospital; Miss A. Lyons, 30 nurses, Children's Hospital; Miss E. Lyons, 5 nurses, "Red Cross House"; Miss West, 5 nurses; Mrs. Leeson, Westmoreland Government Lock Hospital; Miss Shelley, 9 nurses, the Orthopædic Hospital of Ireland; Miss Julia Powell, 3 nurses, Charlemont Street Hospital; Miss Agnew, 2 nurses, Convalescent Home, Stillorgan; Mrs. Burton, 25 nurses, Dublin Nursing Institution; Miss McGiveney, 50 nurses, Mater Miserecordiæ Hospital; Miss Kelley, 55 nurses, Dr. Steevens Hospital; Miss Porter, 9 nurses, Drumcondra Hospital; Miss Tierney, 30 nurses, Nurses' Home, Blackhall Street; Miss Egan, 24 nurses, Coombe Hospital; Miss Brady, 28 nurses, Jervis Street Hospital; Miss Rae, 40 nurses, Cork Street Hospital; Miss Wall, 3 nurses, St. Mark's Ophthalmic Hospital; Miss Shuter, 22 nurses, the Royal City of Dublin Hospital; Miss Ramsden, 36 nurses, Rotunda Hospital; Miss Hosford, 3 nurses, the National Eye and Ear Infirmary; Miss Hampson, 14 nurses, Hospital Portobello; Miss Bradshaw, 12 nurses, the Royal Hospital for Incurables; Miss A. McGiveney, 12 nurses, the Children's Hospital, Temple Street; Miss Law, 2 nurses, Stewart Institution, Palmerston; Miss Spratt, 4 nurses, Nurses' National Lying-in Hospital, Holles Street; Miss J. Powell, 4 nurses, Mercers' Hospital; Miss Violet Roberts, 25 nurses, private nurses; Miss Fitzpatrick, 62 nurses, the Adelaide Hospital; Miss Campbell, 40 nurses, St. Vincent's Hospital; Miss Hughes and Miss Burns, Richmond Asylum.

### The Ceremony.

"One Who Was There" writes: Thursday was a most lovely day, and we each donned our "best bib and tucker" in the shape of uniform, in pleased anticipation of the honour of being received in our official capacity by the Queen. We drove in various vehicles ranging from a carriage to the humble jaunting car through our beautiful and spacious Phœnix Park, of which we are so justly proud, to the Viceregal Lodge. When we had all assembled (a truly noble and dignified gathering, which would have made any poor probationer shiver in her shoes) we were received in the vestibule by Sir Arthur Bigge, private secretary, whose unvarying courtesy in looking after our comfort throughout our visit was most marked. We saw the preparations being made for Her Majesty's afternoon drive. A baize-covered platform is put slanting down the steps, and the Queen is wheeled by her Indian attendant in a chair with rubber tyres to the very door of her carriage. We were then asked to form up in a single line on the gravel sweep in front, headed by Miss Huxley, of Sir Patrick Dun's Hospital (the senior matron of Dublin), who had acted as chairwoman at our meetings on the subject of the address. Next her stood Miss MacDonnell, of the Richmond Hospital, our treasurer; and Mrs. Kildare-Tracey, of the City of Dublin Nursing Institution, our secretary.

Some of us were exercised in our minds as to whether a curtsey or a "bob" would be right on the occasion, but we decided in favour of a "bob" as being supposed to be now the correct thing, and as Her Majesty drove at a walking pace down our line she bowed to each of us, and we "bobbed" as gracefully as we could, thinking regretfully of the old-fashioned curtesy, and of how much prettier it would have looked.

Sir Arthur Bigge then presented Miss Huxley, and the Queen drove off. She was accompanied by H.R.H. Princess Henry of Battenberg and attended by Lady Southampton. We were disappointed not to have seen Princess Christian on the occasion, but she was absent at another function. We did not cheer, as one newspaper erroneously reported, as it would have sounded very feeble indeed. Sir Arthur then led us through the Lodge to the private gardens, which looked very pretty, with the Dublin mountains in the distance, and formed a nice background to our photographed group, as a photographer had arranged to take us there. Some of the matrons did not wear uniform, either from choice or necessity, as certain institutions object to their matrons and nurses wearing outdoor uniform, notably fever hospitals for obvious reasons. This rather marred the general effect of our appearance, and those of us who were in uniform felt that we rather "scored" when the photographer requested those in mufti to stand at the back. Some wore primroses in honour of the day, others elected to wear loyal badges, and one lady had the order of St. John of Jerusalem. We then returned through the drawing-rooms, where we examined, at our leisure, the various photos in frames of the Royal Family and others lying about, which the Queen always carries with her. Her knitting (evidently soldiers' caps) was lying in a work-basket. In one room were scattered about some lovely specimens of Irish lace, and samples of Donegal carpets, brought for inspection. After tea we were shown various objects of interest, tapestry, &c., and returning to the hall we saw the Queen's wheeling chair, and some of the most lively of us (of course, with permission) seated ourselves for one moment on the chair so recently occupied by "the first lady in the land." We then took our departure, after first signing our names in the Queen's visitors' book, as requested, having spent an enjoyable and memorable afternoon.

### The Jubilee Nurses.

On Thursday morning the Queen's reception of the Jubilee nurses took place—a very short and simple function. Fifty of the nurses, in their neat, plain uniform of navy blue cloaks and bonnets, with white collar and strings, were drawn up on the lawn in front of the main portico of the Viceregal Lodge. Her Majesty then appeared from the side of the building, driving herself in her little donkey chair, and passed slowly down the line of nurses, bowing her head and smiling pleasantly, but saying nothing. Each nurse curtsied as the Queen passed her. The Sovereign drove round to a side door out of sight, and the nurses, after being photographed, were entertained to luncheon in the Viceregal Lodge. The Earl of Meath, Mr. Robert O'Brien Furlong, Mr. Luke Teeling, and Mrs. Power Lalor accompanied the nurses during their visit.

### A Royal Function at Donnybrook Hospital.

On Friday Princess Christian inspected the Donnybrook Royal Hospital for Incurables, of which Miss Bradshaw is lady superintendent. The hospital has 206 patients at present. It stands in 14 acres of beautiful grounds, and is made as pleasant and homelike as possible, each patient having a separate cubicle, and entertainments being often organised. A large proportion of the inmates are incurable cancer cases. The cancer wards have small, separate, enclosed rooms at the far end of each, into which patients are removed when the end is so near as to cause distress to the other inmates of the ward.

A Crimean veteran who is one of the patients was amongst those specially noticed by their Royal Highnesses, also a woman who has been in the hospital for no less than 57 years. Princess Christian inspected all the arrangements, and expressed great approval of their comfort and completeness. The hospital and grounds were very tastefully decorated with flags and ornamental poles.

### The Night Nurses' Wail.

"Lamp oil!" "Lamp oil!" It's a good thing in its way,
Save when we hear it shouted out full fifty times a day;
Our hearts are sore within us, and we wish it far away!

The "Home" is all we could desire, and of comfort has no lack,
But that dreadful little noisy street that lies just at the back—
Oh! that business with those lively folks was a little bit more slack!

Of restful hours we need, not only one, but many.
Hark! "Lemons!" "Lemons!" "Lemons!" "only three a-penny!"
"Take away your basket, child, indeed we don't want any!"

A well-played cornet solo is something very nice,
Except when trying to get some sleep at almost any price;
"If we'd but bought a lemon we could stop it in a trice!"

From Saturday to Sunday the noise and din are more,
On Sunday all the church bells ring—there seem to be a score;
And Saturday the children play in a mad and wild uproar!

If "music soothes the savage breast," then civilised are ours,
It only makes us rave and weep, when in the passing hours
A barrel organ stopping near plays, "Buy my pretty flowers?"

"Milk!" "Salt!" "Sandbags!" "Chalkstones!" "Good pears, tuppence a-pound!"
"Oranges!" "Onions cheap to-day!" This is the daily round,
With other wretched hawkers' calls which in our ears do sound.

So we may not, cannot, do not sleep, however we may try,
No "Nature's great restorer" comes to close each tired eye—
We are only yelled at, screeched at, screamed at, importuned to buy!

"Matches!" "Mint, hap'ny a bunch!" "Bootlaces, penny a pair!"
Thinking about the "Land of Nod," and yearning to be there;
We wonder *where* we can get sleep, and *Echo! Answers!* where?

A. G. LUCAS.

### Where to Go.

ST. JAMES'S HALL, PICCADILLY.—Thursday, May 3rd, three p.m., annual meeting of the Zenana Bible and Medical Mission. Lord Kinnaird in the chair.

### OUR CONVALESCENT FUND.

WE have to acknowledge the following contributions to our Convalescent Fund: Nurse Cecil, 5s.; Nurse S. B. M., 5s.; Nurse Beatrice, 2s. We have also received a very grateful letter from a nurse to whom an advance was made, and who returned the money which was lent her for a visit to the seaside.

### Appointments.

CONVALESCENT HOSPITAL AND HOME FOR CHILDREN, MOSELEY HALL, BIRMINGHAM.—Miss Alice M. Armstrong has been appointed Lady Superintendent. She received her training at Brownlow Hill Infirmary, Liverpool. Subsequently she worked under the South London District Nursing Association, then as ward sister, and afterwards as home sister at the Infirmary, Birmingham. At present she is assistant matron at the Park Hospital, Hither Green.

ABERGAVENNY COTTAGE HOSPITAL.—Miss Cecilia E. Hopton has been appointed Nurse Matron. She was trained at the "Mary Adelaide" Nursing Association Institute. Subsequently she was sister and on the private staff the Infirmary and Children's Hospital at Kidderminster. She has lately been engaged in private nursing.

BOLTON INFIRMARY AND DISPENSARY.—Miss Elizabeth Hodgkinson has been appointed Matron. She was trained at Bolton Infirmary, and has been theatre and out-patient sister one year, and sister of the "Mallett" Male Ward for six and a-half years in the same institution.

### Minor Appointments.

EXETER SANATORIUM.—Miss Eva Ryan has been appointed Staff Nurse. She was trained at Milton Hospital for Infectious Diseases, Portsmouth.

### Presentations.

DARTMOUTH AND KINGSWEAR COTTAGE HOSPITAL.—The matron, Miss Hibberd, was on Wednesday last presented with a purse of sovereigns as a mark of appreciation from several subscribers and "grateful patients" for the very excellent services she has rendered during her stay at Dartmouth, and for the very many improvements she has been the means of effecting. The presentation was made by Miss Eva Tew (daughter of the late highly-respected and esteemed chairman), on behalf of the subscribers, and was accompanied by a very interesting letter, with names of subscribers, &c. Great regret has been expressed by very many at Miss Hibberd's leaving, and she carries with her the best wishes for her future welfare and success.

### Hints for Nurses.

### SCRAPED BEEF.

FOR use when only the poorer parts of the meat are obtainable. Cut a piece of steak from the round, about half a pound in weight and about an inch thick. Lay it on a clean meat board, and with a sharp knife scrape off the pulp until there is nothing left but stringy fibre. Season the scraped pulp with salt and make it into small cakes. Broil for two minutes either by direct heat over a clear fire or by heating a clean pan or plate, and, when hot, placing the meat on it. Have both sides cooked sufficiently. This is a safe way for a patient to begin taking solid food. Scraped beef may be prepared very easily over an alcohol lamp.

### Death in Our Ranks.

OLD Crumpsall nurses will learn with regret of the death of Miss E. G. Hanan, late lady superintendent of Crumpsall Infirmary, who died last Saturday night. Any communications should be made to M.P., 137, Kenny Street, Tonypandy, Rhondda, South Wales.

### Scenes in a French Hospital.

By a Correspondent.

I was in France last winter, and there caught scarlet fever. I had gone to a provincial town in order to be English répétitrice in an Ecole Normale d'Institutrices, a position in itself not devoid of "experiences"; but this is irrelevant, except in so far as it explains why I was hurried with all speed from under my adopted roof, and found myself within hail of no relations, and no friends of more than three months' standing, in the Pavillon d'Isolement of the Town Hospital.

A French hospital combines in some respects the functions of a workhouse and of a hospital proper; that is to say, while one part of the establishment is called the Hotel Dieu, and treats diseases of all kinds either at the patient's own expense or at that of the town, there is another, the Hospices, where indigent people, who are in any way helpless, are received more or less permanently. To the Hospices belong the wards of the vieillards, the incurables, the idiots, and the children. The last is the far-famed Crêche, from which, in cases where the parents, because of death, disgrace, or imprisonment, are unable to assist their progeny, stream forth, as soon as they are old enough, the "enfants assistés." They go to the families of peasants in the country or of ouvriers in the towns, each with a little packet of clothes which the adopted parents come to renew every year. There were twelve hundred children thus placed in families at the expense of the Department at the time I was in the hospital, and I saw their twelve hundred packets waiting on yards of shelves in a room adjoining the Crêche buildings.

### The Crêche.

It is pleasant, too, to see the resting-place for these little birds of passage. Imagine a long room with windows at regular intervals, as in a hospital ward, and containing about sixty feet of polished parquetry; this, divided into three compartments by glass partitions, over which in the night curtains of clear muslin are drawn; while further tent-like erections of gauze and muslin protect the doll-beds of the tiniest children against draughts and flies. The beds themselves are all of polished brown wood, with coverings and curtains more like a mixture of sky and cloud than anything else I have ever seen. The amount of washing needed to preserve that delicate and diaphanous look must be simply enormous. Children have a trick of worshipping their beds. I know that in my mind there used to be a quaint connection between mine and heaven; and really to each of these little crêche children their bed must have been suggestive of the spreading wings of

> "Angels in blue and white
> Crowned on the head."

There was a small statue in pink and white and gold of the "Petit Jésus" in the middle of each room, and the whole ward, with its walls of glass and gauze and muslin, gave a curious impression of transparency and seclusion and innocence.

### The Patients' Quarters.

My quarantine, however, was passed among very different surroundings, and until its conclusion brought me liberty to make the round of the hospital I saw nothing but one long sombre dark-green room. Here I had some queer times. Each hospital in France is head-quarters to a branch of a community of non-cloistered *religieuses*, to whom the charge of the nursing is entrusted. These, in taking their vows, have chosen the life of active devotion rather than the more purely contemplative one of the cloister; but from intercourse with the world those I met were almost as far removed as any cloistered nun. They only went out of the hospital at rare intervals to see their family or to fetch patients, and indoors they saw only these and the doctors. I wonder what an English nurse would do without her days off?

### Sœur Anastasie.

Therefore I was surprised to find in Sœur Anastasie, who nursed me in scarlet fever, a cheerful little old woman possessing a large sympathy with the foibles of youth, and a huge capacity, which she lavished on me, for spoiling her patient. She always seemed to me to be a recluse by nature rather than in obedience to the rules of any order. Her narrowness proceeded from natural simplicity, her toleration from natural kindness; a combination which had nearly succeeded in strangling a natural sense of humour which must once have been vigorous. I showed her an illustration in the *Sketch*, called "A Hardy Race," and representing the condolences of the village curate with a hale sexagenarian, who has, if I mistake not, "been walloped by dad for chucking stones at grandfeyther." "Ah," said the little Sœur, "Voila ce que j'aime. Le bonhomme qui console l'autre. Il fait l'œuvre du Bon Dieu." And long after my stammering French had given up trying to make her see the real pith of the matter, she remained gazing in admiration at the clerical collar and big umbrella of "le bonhomme qui console l'autre!" She did not like the photos of actresses and danseuses in my illustrated magazines.

### A Skirt Dance in the Hospital.

But, on the other hand, some time afterwards she and the doctor just succeeded in saving a little child ill with croup; it was a long business, and when nothing remained of my own malady but contagion, the little girl was still in a wearisome convalescence. So one night Sœur Anastasie and I put our heads together to find some way of amusing her. We opened the doors between the "Throat" and "Fever" wards of the Pavillon d'Isolement, disposed the rays of two candles to the best advantage, and in that garish light I danced. First fragments of old step and skirt dances I had learned some six years back, then the Gondoliers cachuca, spurious reels and minuets and Highland flings, and other capers of doubtful sedateness; and that was not all, for Sœur Anastasie played on a sort of musical box we have known in our childhood, called a "Revotina," and containing five notes written in no known intervals of the gamut, no extant measure of time, and which plays backwards and forwards according as you twist it to right or left. If she played faster I danced faster, not to be left behind, faster still if I lagged, to encourage me; and finally she began to hop seriously, first on one foot and then on the other, I the while almost helpless between panting and laughing, but bound to continue because of the pity it would be to stop that grave jig—and the child smiling as she had not done for weeks. Dear little Sœur Anastasie! Her duties were to keep her rule and care for the sick; but if care for the sick included dancing she was ready to do that too; and I liked her for it. It was the final touch to her self-abnegation.

### The Infirmières.

Besides the *religieuses* there are, to do the rough work of the wards, the infirmières, a set of beings much resembling, at my hospital, the race of charwomen among the English. They were some of them taken from among former patients whose illnesses had mentally or bodily handicapped them for the search of employment elsewhere. They were remarkable for doing everything the longest way round, and for an absence of knowledge on most points, notably hygiene. Mine was, however, able-bodied—an excitable, passionate Bretonne, who could not write, and disposed of snuff in large quantities. She took no interest in me after she found I took none in the state of her tabatière. It was she who, when one day I

was explaining some English words to the Religieuse, said, "Ah ! c'est comme ça, alors, que vous dîtes dans votre *patois?*" For her, no doubt, there existed the French language, and a few insignificant idioms spoken in such corners of the earth as Germany, Italy, Russia, and Great Britain, and designated collectively "les patois !"

I must not forget the doctor, a delightful man, who spoke, had he known it, an appreciative word for the hockey and bicycling of *nous autres* by his many compliments on my "si excellente constitution," and his regrets that more Frenchwomen did not possess a similar one.

### The Hospital.

The hospital covers a magnificent area, the different wards being in fact separate buildings, or "pavillons," most of them with well-kept gardens. But contagious patients were apparently not considered to need floral distractions, for in front of my pavillon, between it and the main buildings, was but fifty yards of barren ground; beyond this, on the left the mortuary and dissecting theatre, in the centre the idiot ward, on the right the incurables. Here I passed forty-three days, in company with a varying number of other patients, mostly the sick poor of the town, who were very nice to me, though dull at times. I used to wait for the post half the day; I read what it brought; I wreaked my vengeance on fancy work, which I hate much, and cobble in proportion; and I produced a quantity of patriotic poems on brown paper, which were lost to posterity in the disinfection stove. On March 17th my deliverance came. I thought —in two connections—of the French of Kimberley, and went to stammer polite farewell speeches to my doctor. "Madamoiselle, ça c'est guéri tout seul," he said. "Vous avez une si excellente constitution." Toujours cela! But it is quite true: so perhaps I am not a very good testimonial to my French hospital.

### The French Nursing System.

Not attempting to compare the merits of the two, the French nursing system is necessarily very different from ours. It is far rarer in France than in England for educated women other than *religieuses* to enter the nursing profession. In France an educated woman who finds herself poor (this is putting aside professeurs and institutrices), if she does not get married, will often stay at home and do without servants, where an Englishwoman would prefer to get an occupation and live less simply. The class of workers from which our nurses are formed does not abound in France. Nor, again, is there much opening for such nurses in the hospitals—even if they remained subordinate to the *religieuses*; for the infirmières who occupy this position are seldom asked for skilled labour, and have most often but the status and pay of bonnes. The increase of illness during the last few winters has, however, demanded fresh nurses, and a school has been started in Paris where women, not religieuses, and of a certain amount of education, are trained for the nursing of invalids in their own homes.

### To Nurses.

We invite contributions from any of our readers, and shall be glad to pay for "Notes on News from the Nursing World," or for articles describing nursing experiences, or dealing with any nursing question from an original point of view. The minimum payment for contributions is 5s., but we welcome interesting contributions of a column, or a page, in length. It may be added that notices of entertainments, presentations, and deaths are not paid for, but, of course, we are always glad to receive them. All rejected manuscripts are returned in due course, and all payments for manuscripts used are made as early as possible at the beginning of each quarter.

### Everybody's Opinion.

**[Correspondence on all subjects is invited, but we cannot in any way be responsible for the opinions expressed by our correspondents. No communication can be entertained if the name and address of the correspondent is not given, as a guarantee of good faith but not necessarily for publication, or unless one side of the paper only is written on.]**

### THE ONE THING NEEDFUL.

"Sister" writes: I quite agree with "Nurse Mary" that all institutions should have daily prayers in the wards, especially infirmaries, where the greater number of poor patients have sadly neglected their past lives. But I certainly do not agree with her on the point that it is not allowed on account of Roman Cathclics. During my experience the priest is often looked upon as an intruder, and, as the saying is, has to "take a back seat." As to Roman Catholic patients objecting, it is much the reverse. All those I have come in contact with always feel it deeply that their fellow-creatures so often die without one word about the future ever being spoken to them, and I have frequently known Roman Catholic patients to pray for those dying around them not of their own creed.

### THE AGE LIMIT.

"W. B." writes: Like "Pavo," I have felt that others with a more ready pen would thank Miss Gardner for her letter and common-sense way of dealing with the subject. It is perfectly absurd to fix the age limit at 35, and I feel inclined to say, "or any other," as recent testimonials and certificates have always to be produced. A nurse should be judged by her capabilities and tact. Certainly it seems reasonable that the younger the applicant, combined with length of training, the better the nurse; but some women would not make good nurses if they were trained a dozen years, and it is a pity that such women take up nursing, when perhaps they might excel in some other branch of usefulness. I know several instances of ladies, educated women, who have not had an opportunity of being trained before 28 or 30, who have set their shoulder to the wheel, gone successfully through their training, giving entire satisfaction to their matron and committee, taken up district nursing, and are more alert, energetic, reliable in emergencies, professional in their conduct, and can endure fatigue better at 40 and over than many another at 30 or under.

### ALEXANDRA NURSES.

"A Friend of Tommy Atkins" writes: Not very long ago a most interesting account of her work, by an Alexandra nurse, was published in The Hospital. I should be glad if some more of these nurses would write and tell us what kind of work they do, the rank or position they have in the garrison, and the kind of "quarters" allotted to them. If they will do this it may be a great benefit to other nurses. Last summer a Queen's nurse applied to the S. S. F. Association for a post which was advertised in the columns of The Hospital. She was given a copy of their rules (rules which could only apply to district nurses), and asked if there was anything in them that she objected to. She replied that they were similar to those she had been working under, and she was quite willing to accept them. When she arrived at her destination, an island about 9,000 miles from England, she found that she was expected to do, not district, but private nursing amongst people who were both able and willing to pay for her services, and was given a totally different set of rules to those she had been given when engaged. In the rules for Alexandra nurses it states: "The services of the nurse are in all cases *free*." Most nurses would conclude from that statement that their work would be amongst the poor. Is that nurse justified in breaking her engagement? I think she is, but I should like to know the opinion of other people on the matter. Nurses cannot make too many inquiries before they take posts abroad, as long voyages are very expensive, and if a nurse cannot fulfil an engagement she has to return home at her own expense.

### Wants and Workers.

Cottage Home for Babies.—In answer to a question on this subject by "Certificated Midwife," there is a small unfurnished seven-roomed house to let at £30 a year near Brighton Downs; three good bedrooms, close to a park, and fifteen minutes from sea. Address can be had, if desired, by writing to the office of The Hospital.

### Echoes from the Outside World.

### AN OPEN LETTER TO A HOSPITAL NURSE.

THERE is comparatively no news from the war, although Lord Roberts continues to send despatches detailing the movements of the troops. A grand effort is about to be made to capture the Boers now arrayed in position against us. They wish to prevent our onward march to Pretoria; we want to surround them, close all their lines of retreat, and do a second Cronje stroke on a bigger scale. But it will of necessity take a great deal of strategy and planning to insure success over such an extended field of operations. Consequently, the censor will allow hardly any but official telegrams to come through, and the Bovril Company, who used to issue daily bulletins, have had to give up the experiment for want of news. General Brabant is within eight miles of Wepener, and therefore one of the papers states "Wepener practically relieved," but remembering that exactly the same thing was said about Mafeking two months ago, the statement must be accepted with several grains of salt. Meanwhile, notwithstanding that the only bread obtainable is made of oats and is full of husks, the poor little town had not surrendered on April 9th, though the absence of the name of Baden-Powell in the message makes one a little fearful lest Boer reports should for once have been correct.

WHAT a charming man the King of Sweden and Norway must be! The account of his visit to the Scandinavian Sailors' Home, close to the West India Docks, on Sunday reads like an extract from the "Arabian Nights" entertainment rather than a formal ceremony of 1900. His Majesty, who had attended morning service at the Swedish Church, went straight on to the Rest, and in the little speech which he addressed to the sailors to tell them how glad he was to revisit them, he evidently spoke under the influence of the words he had just heard. This little speech was the only conventional part of the proceedings. The King walked in and out among the sailors, frequently putting his hand on the men's shoulders as he chatted, and proving how excellent a linguist he is by conversing with apparently equal ease in Swedish, Norwegian, Danish, Finnish, German, and English. He was evidently proud of the fact that he is an old sailor, having joined the navy when eleven years old, and thoroughly inspected the whole building, arriving at last at the large dining hall, with its plain long wooden tables, the white cloths set out all ready for the sailors' mid-day meal. "Bring in the dinner," said his Majesty, "and ring the bell for the men." The order was carried out, and then, instead of standing and looking on at the meal, he sat down in genuine homely style at one of the tables and made a hearty meal, not only delighting the men, but cleverly ascertaining for himself that the fare provided was good and ample. No wonder that when he left the hurrahs were almost deafening, as his fellow guests endeavoured to show their enthusiasm and their loyalty.

MONDAY was St. George's Day, and also the birthday of William Shakespeare. We have never been accustomed to make much of the date devoted to the Bard of Avon, except in the town of Stratford; therefore there was nothing extraordinary in the fact that Londoners took no notice of it, save that a basket of white flowers, sent by the Shakespeare Society, and a wreath of lilies from Mr. and Mrs. Benson, were placed before his statue in Poet's Corner. But that St. George's Day was so little observed has set folks talking as to whether our national day of celebration had not better be held in June and called "Empire Day." In the City, especially round the Stock Exchange, there were plenty of red roses to be seen, though complaints were made that the supply was short. The price varied from 3d. to 6d. a bloom. Little red and pink rosettes, supposed to represent England's emblem, could be purchased for the humble copper. The West End, especially Clubland, was gay with flags, in many cases representing the Red Cross of St. George, and Liverpool celebrated the event with great enthusiasm. But in London generally and the suburbs the man in the street failed to evince any special interest. While the green was conspicuous on St. Patrick's Day on horses' heads and in workmen's buttonholes, neither a red badge nor the national colours obtained anything like the same universal use this week. It is hinted that John Bull resents his patron saint being shared with other countries who claim St. George as one of their heroes; but a more reasonable explanation of the difference is that our enthusiasm had worn itself out in the three commemorations which have lately followed each other in such swift succession.

THE Duke of Argyll began his political career so young that to many of us it comes as a surprise that when he died he had not quite completed his 77th year. He had been three times married, and the present Duchess has, it is said, proved herself an excellent and skilful nurse. The Queen alludes to the Duke as "a personal friend of 50 years' standing"; in Scotland, so important was the position which he held, he was often alluded to as "the Duke" simply, with no further amplification. It will seem strange to speak of the Princess Louise as the Duchess of Argyll, yet it is such a grand old title, dating from the thirteenth century, that even a Queen's daughter will be proud of it. Probably as proud as her husband, who, it is well known, could have been a peer before his father died, if he had so desired. But he refused the proffered honour, always remembering that some day he would be Mac Cailean Mhor, the Chief of the Campbells, a title which he values above all others.

IT is a truism that people who live in London know so little of its show places in comparison to our country cousins. I suppose this arises from the fact that they look upon sight-seeing as a duty, whilst we, knowing that we can go any day, always put off the visit till a more convenient season. One day last week I took some children to Kensington Palace to see the former home of their Queen, and came away quite as pleased with my visit as they were. The day was as hot as July; the trees in the Long Walk in Kensington Gardens were just breaking out into early green; the sun shone brilliantly on the gleaming water, where floated fairy boats of all sizes and shapes, launched by eager schoolboys; and the lofty and well-aired galleries gave both refuge and recreation to hundreds besides ourselves. One nurse, and one only, I saw amongst the throng, and I thought I would remind you of the fact that every day but Wednesday the Palace is open to the public free, with plenty of benches to rest upon when limbs are weary. Of course, the Queen's toys came in for the lion's share of attention from the children, and the dolls' house, with its two big rooms, a kitchen, and a dining-room —evidently the Queen's dolls never slept—always had a big crowd round it. Amongst the other toys I was most interested in was a carriage for the dolls, a genuine old yellow chaise, with a rumble similar to the one the Princess generally rode in herself, I expect. The dining-room where the young girl often dined alone with her governess was very small; her sitting-room, on the contrary, being a large room. We only saw the chamber where the Queen was born from across the quadrangle, because it is not supposed to be open to the public; but, as a matter of fact, it is simply a question of payment if you want to see it. One of the uniformed officials informed us in a mysterious whisper, after a little preliminary conversation, that "if we would very much like to inspect the interior of the room, which is almost as it was 80 years ago, he might possibly be able to persuade the people in charge to accord us the privilege." He was obviously disgusted because I declined; but as I observed him subsequently conduct three or four parties through the private door, I concluded that the "people in charge" did not require a vast amount of persuasion.

### Cookery for Invalids.

### FOOD FOR THE ALBUMINURIC.

For some severe cases of Bright's disease an entire diet of milk is sometimes ordered and found very beneficial, such as six ounces every hour and a glassful night and morning, in all about three or four quarts per day. After, perhaps, some weeks of this severe diet the return to ordinary food must be very gradual. At first the milk should be thickened only with a little arrowroot, then very thin milk puddings made with rizine, tapioca, or sago, followed by light puddings, before the patient has fish, chicken, or vegetables.

Solid butcher's meat must be steadily avoided, but consommé made from beef bones may be given, and also used as stock for the vegetable soups which are so excellent for this class of patient.

But much depends on the meals being not only nutritious, but attractive in every way, because sufferers from this trying disease have but a poor appetite as a rule, a weak digestion, and constant feeling of nausea; these are in extreme cases. In slighter ones just as much care should be taken, as want of this may aggravate the disease.

Consommé.—I know no better recipe than this, as it combines the uses of the bones, meat, and vegetables, and is most delicious to the palate. Put a shilling's-worth of fresh meat bones (well broken) into a large saucepan and cover them entirely with cold water; bring gently to the boil, add a little cold water and remove all the scum, then add a good pinch of salt, six peppercorns, two large carrots, four onions, half a stick of celery; stick four cloves in one onion, add a bunch of herbs (such as thyme, parsley, and bayleaf). Simmer all for a few hours, adding a little more water occasionally. Strain off through a hair sieve and leave till the next day, when remove every particle of fat. Measure the stock, and if there is a quart allow half a pound of lean neck of beef and four whites and shells of egg to clear it. Chop the meat finely and put it in a clean stewpan with the whites of eggs. Stir it well, add the quart of stock, a few slices of carrot, one leek, a piece of celery, two peppercorns, and any cooked poultry bones. Bring the stock gently to the boil, stirring it occasionally. Then simmer for one hour and strain through a soup-cloth or clean serviette that has been wetted in hot water and tied over a basin. To re-heat the consommé put as much as is wanted in a jug and stand this in a saucepan of boiling water.

Thick Lettuce Soup.—Put one ounce of butter in a stewpan with one sliced Egyptian onion, a bunch of herbs; cut up a pound of well-washed and dried lettuce; add this, cover the stewpan, and cook the lettuce for fifteen minutes. Then stir in one ounce of ground rice and a pint and a half of fresh milk; cook for thirty or forty minutes, then rub all through a hair sieve. If there is a quart of purée, put it in a stewpan, and when hot stir in the raw yolks of two eggs, half an ounce of butter, a dessert-spoonful of lemon juice, and three tablespoonfuls of cream, and a little salt. Stir till it thickens, but on no account let it boil. Add a little sap green to make it a good colour, and strain it into a hot tureen. Fried croûtons can be served with it if liked, but they must be on a separate dish.

Vegetable Soup.—Take one large carrot, one onion, one peeled potato, one turnip, one leek, a handful of lettuce, and bunch of well-washed and picked watercress; cut them up and fry in one ounce of butter. Add a dessert-spoonful of ground rice, mix well, and stir into the vegetables one and a half pints of milk. Let all cook gently till tender; then rub all through a hair sieve. Put the purée into a clean stewpan, add a little cream, the raw yolk of an egg, a piece of butter, season with salt, and stir till the soup thickens; serve croûtons with it.

Macaroni.—Macaroni will make a variety of dishes, and is considered very wholesome for an albuminuric. Made in the following manner it is a delicious savoury. Put two ounces of the best macaroni into plenty of boiling water in a saucepan, adding a teaspoonful of salt; boil for twenty minutes, when it should be quite tender; strain it and wash well in cold water, then cut it into short lengths and put it in a buttered pie dish. Put three tomatoes in a saucepan with half an ounce of butter and a little water, cook them on the stove for a few minutes, then rub the tomatoes through a sieve. To this purée add a little more butter and one ounce of grated Parmesan cheese; mix together and pour over the macaroni. Stand the dish in a tin containing hot water and bake in a hot oven for a few minutes.

Purée of Cabbage and Potato.—These make a very good combination. Boil two or three potatoes and a young cabbage in salted water, rub the potatoes through a sieve, press all the water from the cabbage; chop this and rub it through also, then mix the two purées together in a stewpan, add one ounce of butter and a little cream and pepper and salt; make all quite hot, then dish up in a pile and put some fried dice of bread round.

Tomatoes with Mushrooms.—Chop a few peeled mushrooms finely, also a small piece of onion, put both in a small saucepan with half an ounce of butter, and fry for eight minutes; mix in a dessert-spoonful of bread crumbs and a little chopped parsley and pepper and salt. Scoop out the pips from two or three tomatoes and fill them with the above mixture; on the top put a few browned bread crumbs; stand them in a lightly-buttered tin and bake them for about twelve minutes. Dish up on rounds of toast, cut with a round cutter about an inch and a half in diameter.

Egyptian Onions.—These are now the best in the market, and are excellent cooked in this way: Peel them and put them to boil in salted water; as soon as they do pour off the water, wash them in cold; then put them into plenty of boiling water, season with salt, and cook gently for one or more hours according to size. When tender take them up on a sieve, place them in a hot dish, and put on each onion a piece of maître d'hôtel butter, made by mixing together half an ounce of butter, half a teaspoonful of chopped parsley, and the same of lemon juice.

French Beans with Cream.—String and cut one pound of French beans; plunge them into boiling water that is seasoned with salt and a tiny piece of soda, boil for fifteen or twenty minutes, then drain in a colander. Put one ounce of butter and half an ounce of flour into a stewpan and fry them together, mix in a quarter of a pint of cream and stir till it boils, add a teaspoonful of lemon juice, and season with pepper and salt. Put in the beans, reboil, and turn out on to a hot dish. Garnish with little rings of bread fried a nice golden colour.

Compôte of Fruit.—Make a syrup by boiling together till clear half a pound of sugar and a quarter of a pint of water. Take a quarter of a pound each of different kinds of fruit and cook them for a few minutes separately in the syrup, keeping them in separate basins when cooked. The same syrup will do for all. Great care must be taken not to destroy the fresh flavour of the fruit by overcooking it. When cold, arrange the fruits alternately in a glass dish, and pour the syrup over them.

Rice Blancmange.—Put one and a half ounces of Carolina rice in a stewpan with sufficient cold water to cover it, bring to the boil, then strain off and wash it in cold water; put in half a pint of fresh milk, with a tiny piece of cinnamon and two ounces of sugar; cook gently till the rice is tender, then mix in five sheets of finest leaf gelatine and four drops of vanilla essence, put it into a basin, and when cool add a quarter of a pint of whipped cream, mix well together and pour into a pretty mould; when set, dip the mould in warm water and turn out into a glass dish.

### A Book and its Story.

### RUDYARD KIPLING ON HIS TRAVELS.*

MR. RUDYARD KIPLING's recently-published book of travel is a collection of contributions to the Indian *Pioneer* and the *Civil and Military Gazette* between the years 1887 and 1889, and as such must be placed among his earlier, and first essays in prose. As a collection the articles contained in "From Sea to Sea" are a series of vivid, picturesque impressions, written with that peculiar fervent, forcible, singularly apt power of expression which makes the author inimitable in the particular style of which he is himself master. Their publication in book form was necessitated by the temerity of publishers, who, "not content with disinterring old newspaper work from the decent seclusion of office files, have in several instances thought fit to embellish it with additions and interpolations," and so Mr. Kipling, in self defence, has given these papers to the public in a non-condensed form, for which it has to thank audacious and enterprising publishers.

It is difficult at any time to dip into books of mark and make extracts. Divorced from the context, a passage loses much of its significance and force. With a writer of Mr. Kipling's individuality the operation is even a more delicate one than ordinary. The first volume deals with India chiefly. It opens at Bombay, the city of the author's birth and where his early childhood was spent. From Bombay to Chicago is a far cry, but the impressions cover ground between those two notable places, and the last spot to which he retires before setting out again in order to efface "the clang and tumult of Chicago" from his brain is the tiny township of Musquash on the Monogahela River. Here "summer was on the orchards, and the apples—such apples as we dream of when we eat the woolly imitations of Kashmir—were ripe and toothsome. It was good to lie still in a hammock with half-shut eyes, and, in the utter stillness, to hear the apples dropping from the trees and the tinkle of the cow-bells as the cow walked statelily down the main road of the village. Everybody in that restful place seemed to have just as much as he wanted; a house with all comfortable appliances, a big or little verandah wherein to spend the day, a neatly stored garden with a wild wealth of flowers, some cows, and an orchard. Everybody knew everybody else intimately, and what they did not know the local daily paper—a daily for a village of twelve hundred people!—supplied." With the exception of the dailypaper and the verandahs this summary of rural delights would apply equally to many English villages, only, as we read farther, we cannot boast a "court house, a gaol, where some most enviable prisoners lived, and four or five churches of four or five different denominations."

In the "sweet land of liberty" Mr. Kipling was struck with the freedom accorded socially to women, while "the American of wealth is 'owned' by his family. His working hours are spent in their service. They exploit him for bullion, and sometimes it seems to me that his lot is a lonely one. The women get the ha'pence; the kicks are all his own." And yet, "they develop greatly when a catastrophe arrives and the man of many millions goes up, or goes down, and his daughters take to stenography or type-writing."

As to patriotism, "the men and women set us an example in this. They believe in their land and its future, and its honour, and its glory, and they are not ashamed to say so. From the largest to the least runs this proud conviction, to which I take off my hat, and for which I love them." But have not recent events at home proved that America nor any other country can give us points on this question, and that the definition of the patriotism of the ordinary Englishman, given in 1889, is quite out of date now. The author adds ironically, "An average English householder seems to regard his country as an abstraction to supply him with policemen and fire brigades." The American sings:

"My country, 'tis of thee,
Sweet land of liberty,
Of thee I sing!"

"I have heard a few thousand of them engaged in that employment. But if there is too much Romeo and too little balcony about our National Anthem, with the American article it is all balcony. There must be a born poet who shall give the English the song of their own, own country—which is to say, of about half the world. Remains then to compose the greatest song of all—the Saga of the Anglo-Saxon all round the earth. For we, even we who share the earth between us as no gods have ever shared it, we also are mortal in the matter of our single selves. Will anyone take the contract?" We will not linger over the Indian letters, for there is little new in these. India as a country is familiar enough to most readers, either from personal knowledge, the information of friends, or from the many books bearing on the subject.

Of Japan Mr. Kipling writes in fascinating language. Through the cabin port-hole he gets the first glimpse of cherry-blossom land. "Two great grey rocks stand out studded and streaked with green and crowned by two stunted blue-black pines. Below the rocks a boat, that might have been carved sandalwood for colour and delicacy, was shaking out an ivory-white frilled sail to the wind of the morning. An indigo-blue boy with an old ivory-white face hauled on a rope. Rock and tree and boat made a panel from a Japanese screen, and I saw that the land was not a lie. . . . I was in Japan—the Japan of cabinets and joinery, gracious folks and fair manners; Japan, whence the camphor and the lacquer and the shark-skin swords come. . . . If you desire details of house construction, glimpses of perfect cleanliness, rare taste, and perfect subordination of the thing made to the needs of the maker, you shall find all you seek, and more. . . . The books have told long ago how a Japanese house is constructed chiefly of sliding screens and paper partitions . . . but all the telling in print will never make you understand the exquisite finish of a tenement that you could kick in with your foot and pound to matchwood with your fists. . . . Then I fell admiring the bloom on the people's cheeks, the three-cornered smiles of the fat babies, and the surpassing 'otherness' of everything around me. It is so strange to be in a clean land, and the stranger to walk among dolls' houses." "Japan is soothing to the short man (the author, perhaps, speaks feelingly); nobody comes to tower over him, and he looks down upon all the women, as is right and proper." A Japanese theatre was, of course, visited. "The interior was almost dark, and there was only standing room beside the Japanese policeman, who with Napoleonic dignity stood with folded arms drawn up to the full height of his four feet eight inches. The building was as delicate a piece of cabinet work as any of the houses; roof, floor, beams, props, verandahs, and partitions were of naked wood, and every person in the house was smoking a tiny pipe." "'It's not exactly safe,' said the Professor, 'but if that curtain catches that naked light on the stage, or you see this matchwood gallery begin to blaze, I'll kick out the back of the refreshment buffet, and we can walk home.' With this warm comfort the drama began. . . . A two-sworded man in black and gold brocade rose up and imitated the gait of an obscure actor called Henry Irving, whereat, not knowing he was serious, I cackled aloud till the Japanese policeman looked at me austerely. Then the two-sworded man wooed the Japanese fan lady . . . and trouble ensued in a Vincent Crummles fight between rival lovers, to the music of all the orchestra." Here we must leave our readers, commending to them "From Sea to Sea" if they care for a book in which the characteristics already named, and those of charm, colour, originality, and humour are conspicuous.

* "From Sea to Sea, and other Sketches." By Rudyard Kipling. (Publishers: Macmillan and Co., London. Two vols., 6s. each.)

### For Reading to the Sick.

"O REST in the Lord; wait patiently for Him."

Shut out from life's fair joys,
From spring's bright flowers;
From all its joyous scenes,
With weakened powers.
Lie a prisoner in my place,
And sing God's grace.
The busy world goes on,
My hands are still;
My feet no longer haste
To do my will.
But I have learned to yield, and idle be
While others ply.
Shut out from home's dear cares
When I would fain
Labour for those I love
With hand and brain.
Shut out from paths my feet erewhile have trod,
Shut in with God.
And Thou has taught me, though to learn so slow
This truth to know,
Whether by fervent toil or lying still,
He only truly serves who does Thy will.
Then where and how Thou wilt Thy servant use;
I would not choose. —*S. M. G.*

Come Thou, O come,
Sweetest and kindliest,
Giver of tranquil rest
Unto the weary soul;
In all anxiety,
With power from Heaven on high console.
Come Thou, O come;
Joy in life's narrow path,
Hope in the hour of death.
Come, blessed Spirit, come;
Lead Thou us tenderly
'Till we shall find with Thee our home.
—*Moutrie.*

**Reading.**

How shall we rest in God? By giving ourselves wholly to Him. If you give yourselves by halves you cannot find full rest; there will be ever a lurking disquiet in that half which is withheld. Martyrs, confessors, and saints have tasted this rest, and "counted themselves happy in that they endured." A countless host of God's faithful servants have drunk deeply of it under the the daily burden of a weary life—dull, common-place, painful, or desolate. All that God has been to them He is ready to be to you. The heart once fairly given to God, with a clear conscience, a fitting rule of life, and a steadfast purpose of obedience, you will find a wonderful sense of rest coming over you.—*Jean Nicolas Gron.*

Well may I rest in Thee, and be still. All the past has been ordered by Thee; all that has brought me to my present state, and my present state itself. Let me indulge in no vain regrets; let me not disturb my mind with wishes that this thing or that had not happened. "Be still, and know that I am God," is what the Lord is now saying to me. I do not see why those troubles came; I do not know the reason for my being laid aside; but let "I am God" be enough. He knows what I do not know; that which I cannot see is all plain to Him. He is working out His own designs, and His designs are perfectly wise and good. Let me lie still. Let my heart echo the twice-repeated words of the Psalmist, "The Lord of hosts is with us; the God of Jacob is our refuge." If I can but say that, then I cannot fail to "be still."—*Bourdillon.*

### Notes and Queries.

THE Editor is always willing to answer in this column, without any fee, all reasonable questions, as soon as possible.

But the following rules must be carefully observed:—

1. Every communication must be accompanied by the name and address of the writer.
2. The question must always bear upon nursing, directly or indirectly.

If an answer is required by letter a fee of half-a-crown must be enclosed with the note containing the inquiry.

*Massage.*

(34) Is there any hospital where I can be trained in massage without paying any fee, and take a small salary?—*W.*

Apply to the Institute of Trained Masseuses, 12, Buckingham Street, Strand. Many dangers attend the attempts to obtain instruction in massage work, and the advice of thoroughly trustworthy people should be taken.

(35) Will you kindly tell me what is the address of the best medical man or woman to study massage under who teaches the Nauheim treatment and Schott's movements?—*St. Patrick.*

See answer to "W."

*Gunshot.*

(36) Can you advise me the best books (and least costly) on the latest antiseptic treatment in surgery? Also on the treatment of gunshot and other wounds? If you cannot advise me on these points, perhaps you could do so through THE HOSPITAL.—*H. C. F.* and *F. H.*

Perhaps one of the following would be suitable: "Manual of the Antiseptic Treatment of Wounds" (for students and practitioners), by Watson Cheyne, price 4s. 6d.; "Miles' Surgical Ward Work and Nursing," 3s. 6d. (the first section, comprising 17 chapters, is on antiseptic surgery); "Stevenson's Wounds in War, 18s. The Scientific Press would procure any of them for you.

*Exercises.*

(37) "F. B." would be much obliged if you would give her name and price of a good book describing suitable exercises to use in disease and weakness of the spine.

"The Relief and Cure of Spinal Curvatures," by P. S. Lewis. Price 4s. 6d.; or "The Treatment of Lateral Curvature of the Spine," by Bernard Roth. But no nurse should attempt to "treat" such cases.

*Homes for Inebriates.*

(38) Would you kindly forward to me the names and addresses of a few of the best inebriate homes in England, Scotland, and Ireland?—*A. T.*

As a full list of the homes would occupy too much space, we must refer you to "Burdett's Hospitals and Charities." We do not recommend individual homes.

*Motherless Girl.*

(39) Will you kindly tell me if there is any institution where a motherless girl of 15 could be received? She is rather deformed and very delicate, but is capable of doing very light house work.—*K. T.*

You would probably find that Miss Janes, 59, Berners Street, Oxford Street, W., could best advise you upon the subject.

*Peptonising Milk.*

(40) Would you kindly tell me how to peptonise the white of eggs for nutrient enemata? Also, how to peptonise raw beef juice?—*Sister Martha.*

Nutrient enemata are peptonised in quantities of not more than six ounces, one or two teaspoonfuls of liquor pancreaticus being added to this quantity, and the enema heated to 100 deg.F., and allowed to digest for two hours. Of course, beef juice when peptonised ceases to be "raw." It is not a pleasant preparation except for enemata.

*Chronic Asthma.*

(41) Can anyone tell me of a home where a young woman suffering from chronic asthma could be received for a permanency at 7s. or 8s. per week?—*Ellen W.*

You will find a long list of charitable institutions, of which some would possibly be suitable, in "Burdett's Hospitals and Charities."

*Crippled Boys.*

(42) Can you tell me of any school for crippled boys in the neighbourhood of Bethnal Green.—*C. C. T.*

There is a school for invalid children in connection with the Passmore Edwards' Settlement, Stepney. The Secretary, the Invalid Children's Aid Association, 18, Buckingham Street, Strand, might be able to give valuable advice.

*Cases Unfit for Women.*

(43) Would you kindly tell me if there is any remedy for preventing people engaging nurses for cases not fit for women to undertake?—*F. D.*

The only remedy is to make all possible inquiry beforehand; and, of course, it is always possible for a nurse to ask for references if she deems it wise. Your experience was certainly a very unpleasant one.

*Standard Books of Reference.*

"The Nursing Profession: How and Where to Train." 2s. net.
"The Nurses' Dictionary of Medical Terms." 2s. 6d. net.
"Burdett's Series of Nursing Text-Books." 1s. each.
"A Handbook for Nurses." (Illustrated.) 5s.
"Nursing: Its Theory and Practice." New Edition. 3s. 6d.
"Helps in Sickness and to Health." Fifteenth Thousand. 5s.

All these are published by THE SCIENTIFIC PRESS, Ltd., and may be obtained through any bookseller or direct from the publishers, 28 & 29, Southampton Street, London, W.C.

### Travel Notes.

### XLVIII.—MALAGA IN WINTER AND SPRING.

Thus far Malaga is but little known to that large section of the travelling public that likes to winter in the South, either from considerations of health, or because of the undeniable joy of escaping the fogs and discomforts of our Northern winters.

### The Journey.

There are several routes by which to reach Malaga; the quickest and most direct is by rail viâ Paris, Irun, Madrid, and Cordova. Fare, first-class, viâ Dover and Calais, £12 12s. 6d.; second-class, £10 11s. 3d.; viâ Dieppe about £1 cheaper. Beyond Irun (the frontier town) passengers with second-class tickets will go on first-class. The most convenient, indeed, the only, express train for Madrid leaves Paris at 10.30 p.m., reaching Madrid at 7 a.m. on the second morning. It is obligatory to remain in Madrid one entire day, as the express to Malaga goes at 8.5 p.m. three nights a week only. It would, however, be a great pity not to spend two or three days in Madrid, where there is so very much to be seen. The cold is intense in winter and early spring, just as the scorching heat of summer is almost equally unendurable, so remember to have all your warmest clothing and hot-water bottles for your beds. A much cheaper way is by sea, and for good sailors the voyage is most enjoyable. If you go by P. and O. steamer you must reship at Gibraltar, and the expense is considerable. By Hall's Line you go straight from London to Malaga for £8 8s. first-class; return tickets, available for six months, £15 15s. Time occupied on the journey ten to twelve days.

### The Climate and Advantages of Malaga.

The chief reason that Malaga has not become so popular a health resort as various places on the Riviera is due to the fact that it is not considered so suitable for cases of pulmonary weakness, though very beneficial to those suffering from chronic rheumatism, gout, and scrofulous complaints. Then the sanitary arrangements leave much to be desired, being primitive to the last degree. How far this may be considered a real danger it is hard to say, when one observes the exceedingly healthy state of the city as evidenced by the mortality statistics. The climate is very delightful, the city lies open to the south and east, and absolutely sheltered from the north winds, and those who, without being positive invalids, would in England be shut up most of the winter and spring, may in Malaga bask in sun and fresh air every day. It is somewhat of a drawback that the town being closely built over, the streets high, narrow, and shady, there is not as much air as one would like, but if rooms are taken in the suburb called the Caleta this difficulty is overcome. The water supply of Malaga is above reproach; it is brought from a spring many miles west of the city in large pipes. The pressure is so constant that no deposits can exist. This is an advantage that can hardly be over-rated; it is such a rare treat on the Continent to be able to drink water fresh and cold without the odious preliminary of boiling it.

### The Question of Finance.

The money exchange is now greatly in our favour. The rate is from 34 to 35 pesetas to our sovereign. A peseta is tenpence. In France the rate is 25 francs (a franc is also tenpence) to our sovereign; so you will see how greatly we gain by the exchange. I was myself in Spain two years ago, and I felt that my usually light purse resembled the widow's cruse of oil. You must, however, be careful not to encumber yourself with an overplus of Spanish money towards the end of your stay, because the loss is proportionately big when ou cross the frontier again.

### Hotels and Apartments.

The best known hotel is the Roma; it has a lift (rather a rare luxury), and the lowest pension terms are ten pesetas. Then there is the Nuevo Victoria and the Nizza. There are cheaper houses than these, chiefly commercial hotels, but the food is good and abundant. There is also a good pension in the Caleta, which offers reasonable terms. With regard to apartments, there are but very few, the custom being more to have a room in an hotel and go out to meals, but this naturally would not commend itself to any but the robust and energetic.

### Objects of Interest.

It has very few art treasures, and is not very rich in architecture, though from an artistic point of view the curious old streets of the ancient quarter are entrancing. The cathedral, which is such a striking object from the sea, loses something of its interest on a nearer inspection. It has gone through many building vicissitudes. The present edifice was begun in 1528, but partially destroyed by an earthquake in 1680; in 1719 it was rebuilt, but even now it remains in an unfinished condition. Close by is the Bishop's Palace and the beautiful Puerta del Sagrario. The rock and lighthouse of Gibralfaro is one of the lions of Malaga; it is extremely interesting, and the Moorish horseshoe arches are well worth seeing. The Alameda, or principal promenade, is very fine. A military band plays here every evening, and very charming it is to saunter up and down and round the lovely sixteenth-century fountain brought from Genoa by Charles V.

### Passports.

It is necessary to have a passport to insure receiving your letters at the Poste Restante. An ordinary foreign office passport should be visé by the Spanish Consul-General, the whole cost being 15s. 10d., 3s. 6d. for the passport and 12s. 4d. for the Spanish visé. Messrs. Gaze or Cook will manage this for you.

### Railway Travelling in Spain.

I think some misconception exists on this subject. You will probably be told, as I was, that it is absolutely impossible to travel anything but first-class. Perhaps twenty years ago it was so, but not now. I did most of my travelling third-class, and found the carriages quite as good as those on the French lines. It is quite untrue that they are dirty or in any way unfit for ladies. The one real disadvantage is the slowness of all trains except the express. It would be most unwise, for instance, to try and make the journey to Madrid third-class; indeed, it could not be done without several breaks after Bordeaux, but short journeys in Spain itself are infinitely amusing by third-class trains. The peasants are civility itself, and have all the graceful dignity of their race, and you learn more of Spanish ways and manners in one such journey than in a week of hotel life. You will, perhaps, be a little surprised to find a soldier with a loaded musket get into your carriage, but it gives one a comfortable sense of protection after the first astonishment. There are always one or two such guardians on every train. I imagine the custom arose from the constant Carlist troubles.

### TRAVEL NOTES AND QUERIES.

Rules in Regard to Correspondence for this Section.—All questioners must use a pseudonym for publication, but the communication must also bear the writer's own name and address as well, which will be regarded as confidential. All such communications to be addressed "Travel Editor, 'Nursing Mirror,' 28, Southampton Street, Strand." No charge will be made for inserting and answering questions in the inquiry column, and all will be answered in rotation as space permits. If an answer by letter is required, a stamped and addressed envelope must be enclosed, together with 2s. 6d., which fee will be devoted to the objects of the "Hospital Convalescent Fund." Any inquiries reaching the office after Monday cannot be answered in "The Mirror" of the current week.

Bay of St. Malo (Tramp).—Very nice accommodation to be had for 5 francs per day in June (not so cheap later) at Madame Pallot, Maison Massias, St. Servan. About the same terms at Miss Humphrey's, 20, Place Constantine, St. Servan. Paramé has better sea beach; it is close to St. Servan and St. Malo, reached by tram. Hotel des Bains, 6 francs, and Hotel de France the same. At any of these places it is well to make arrangements beforehand. Full information as to the locality in The Hospital of May 6th and May 13th, 1899.

## Figures and Tables

**Fig. 6. f1:**
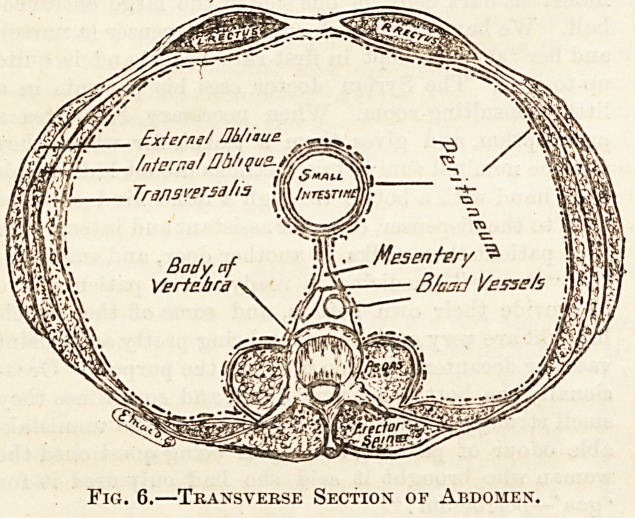


**Fig. 7. f2:**
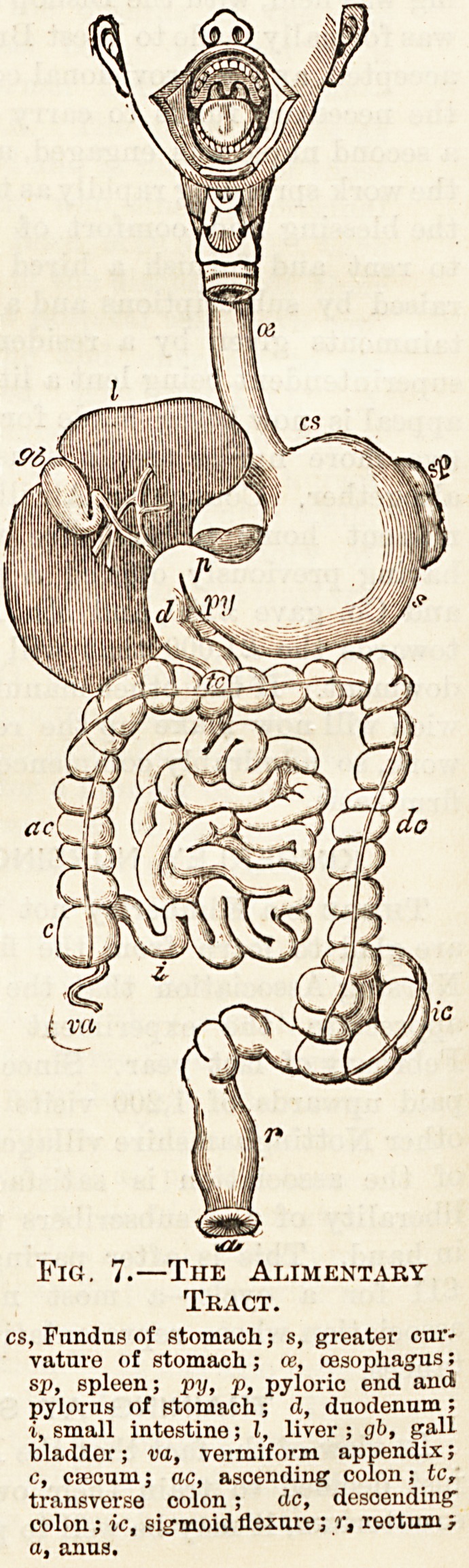


**Fig. 8. f3:**